# Ayurveda and Panchakarma: Measuring the Effects of a Holistic Health Intervention

**DOI:** 10.1100/tsw.2009.35

**Published:** 2009-04-27

**Authors:** L. A. Conboy, Ingrid Edshteyn, Hilary Garivaltis

**Affiliations:** ^1^ Osher Research Center, Harvard Medical School, Boston, MA, USA; ^2^ Kripalu School of Ayurveda, Stockbridge, MA, USA

**Keywords:** Ayurveda, health behavior change, complex medical system

## Abstract

Ayurveda, the traditional medical system of India, is understudied in western contexts. Using data gathered from an Ayurvedic treatment program, this study examined the role of psychosocial factors in the process of behavior change and the salutogenic process. This observational study examined associations with participation in the 5-day Ayurvedic cleansing retreat program, Panchakarma. Quality of life, psychosocial, and behavior change measurements were measured longitudinally on 20 female participants. Measurements were taken before the start of the program, immediately after the program, and 3 months postprogram. The program did not significantly improve quality of life. Significant improvements were found in self-efficacy towards using Ayurveda to improve health and reported positive health behaviors. In addition, perceived social support and depression showed significant improvements 3 months postprogram after the subjects had returned to their home context. As a program of behavior change, our preliminary results suggest that the complex intervention Panchakarma may be effective in assisting one's expected and reported adherence to new and healthier behavior patterns.

### INTRODUCTION

Ayurveda is the traditional medical system of India and, according to the National Center for Complementary and Alternative Medicine (NCCAM) at the National Institutes of Health, it is understudied in western contexts[1]. While there are numerous pilot studies that suggest the effectiveness of Ayurveda to treat specific diseases[2,3], this is the first published scientific study of the Ayurvedic treatment Panchakarma in a western country. Panchakarma is believed to help cleanse the body of impurities that can lead to disease.

Ayurveda is a holistic, complex, medical system. In order to study complex, multidimensional, or traditional medical systems effectively, research needs to begin with descriptive or observational studies first. Without such work, even the best science can yield confusing results[4,5,6]. Initial descriptive work can illuminate theoretical frameworks and elemental constructs that need to be understood in order to formulate controlled studies. Only after such descriptive work, and using observations there gleaned, can we move on to observational trials and, if warranted, controlled clinical trials.

In the fall of 2005, in an effort to better understand Ayurvedic practices, one of the authors (LC) began descriptive work at a school of Ayurveda in Stockbridge, Massachusetts. The school is housed at the Kripalu Center for Yoga and Health, a yoga and Ayurvedic training center. The author began with descriptive observational work, including multiple trips to the site to perform informal interviews with students at Kripalu's school of Ayurveda, and to meet with the designer of the Ayurvedic programs (HG) and staff. The goal was to find a practice best fit to be the subject of an observational trial. The author chose to begin study on one particular Ayurvedic treatment package called Panchakarma[2]. Panchakarma is a relaxation and cleansing program traditionally used to assist with seasonal and social changes (e.g., menopause). The specific techniques used in Panchakarma are described below. This technique was chosen because of its theoretical importance to Ayurvedic medicine, and also because the beginning and ending of treatment are defined, offering a clear pre- and post-test mark for evaluation of effects and gathering of data for a later controlled trial.

Western users of other types of holistic therapies like Ayurveda frequently report improved or strengthened social connection as one benefit of therapy[7,8]. Indeed, holistic therapies, defined as therapies that are designed to treat the individual's body, mind, and spirit, may lead to benefit via psychosocial mechanisms, and the initial descriptive work of Ayurveda at Kripalu pointed to the importance of social factors in healing. Given this, in the design of this observational trial, measures of psychosocial change were included. The goal of this study was to see if this holistic health program Panchakarma, part of the Ayurvedic medical tradition, would show salutogenic benefits, including positive changes in perceived social support, quality of life, and positive health behaviors.

Measurement occurred immediately before the start of the program, immediately after the program, and 3 months following the 5-day onsite therapeutic treatment. Post-treatment, significant improvements were found in comparison to baseline and effectiveness was maintained after 3 months. As a program of behavior change, preliminary results suggest that the complex intervention Panchakarma may be effective in assisting one's expected and reported adherence to new and healthier behavior patterns. The results also suggest that the treatment is effective in altering perceived social support in the short term and potentially in the longer term. Disambiguating which components of the therapeutic program were most essential in driving these changes is necessary and the varied outcomes offer indication as to which were the more effective pieces of the intervention. More importantly, the study of this treatment in its holistic complex form offers insight into the development of effective health interventions more generally.

### BACKGROUND

#### The Mechanism of Social Support

As mentioned above, given the theoretical relevance of social support, and the psychosocial world in Ayurveda and other holistic therapies, we decided to measure social mechanisms of changes. There is much scientific western literature on the therapeutic benefits of social support. High perceived or observed social support has been linked to improved health outcomes in both human and animal studies. For example, an often-replicated finding in both animal as well as human observational and experimental studies is that individuals with low levels of social support have altered immune function[9,10,11,12,13]. Social support may buffer or mediate responses to stress and allow for the maintenance of a healthy immune system. Evidence of this hypothesis has been found through many types of investigations: chronic stress has been reported to alter immune function and cytokine production[14], and to reduce the immune system's response to anti-inflammatory signals[15], the chronic stressor of low socioeconomic status in childhood is inversely related to immune function independent of higher socioeconomic status later in life[16]. Most generally, people with better quality relationships feel valued and are healthier[13,17].

Given the importance of social factors in health, there has been much research attempting to modify or augment social support, especially in high-risk populations, but modifying this factor can be tricky. For example, naturally occurring emotional support appears to improve cognitive resilience following a stroke[18], but not if the additional support is prescribed[19]. This suggests that perceived social support has an emergent quality and perhaps the mechanisms of social change, if they exist at all, require direct modification of the subjects' perception of themselves in their social context. Some health and health behavior change programs contain modifications to social factors implicitly. Ayurveda, like other holistic medical systems, attempts to influence the social world directly by considering the individual in his/her social context. Such programs may offer clues on how to best deliver health behavior programs in order to make them more effective and increase adherence.

There is some preliminary research in this area. A 12-week Tai Chi exercise program that assessed perceived psychosocial status showed statistically significant improvements in all measures of psychosocial status, including perceived social support[20]. Another study that involved an educational and experiential retreat program for cardiac rehabilitation patients found significant positive associations between increased well-being and feelings of increased connection to others[21]. This program involved many elements, including group discussion; educational seminars on nutrition, exercise, and stress management techniques; as well as experiential practices in communication skills, yoga, and meditation. The participants rated their perceived change in sense of connection to others as greatly increased after the retreat. These encouraging findings suggest that other types of holistic and Complementary and Alternative Medicine (CAM) therapies may further substantiate the role of psychosocial factors in improved health and well-being outcomes.

### The Panchakarma Program

In Ayurveda, an ancient Indian medical system, the human body is composed of derivatives of the basic elements of ether, air, fire, water, and earth, and these derivatives must maintain an equal balance within the body to maintain health. The healing and prevention process of Ayurveda differs from Western biomedicine in that it is based on the principle of promotion of health rather than elimination of disease. If balance is disturbed, the Ayurvedic perspective supports that toxins, or "ama", will accumulate and saturate the tissues of the body. This can lead to disease[3]. Ayurveda shares, with other holistic, multidimensional systems, the ability to provide medical care with a healing process that is customized to the patient, maintaining the personalization that is missing in some western medical treatments. As with other holistic therapies, the social world of the patient is considered an active factor in health and disease. Indeed, one definition of holism is that the subject is considered in the context of his/her social world[6]. In Ayurvedic diagnosis, the quality of social relationships and social support are considered important factors in disease causation and treatment plans will be modified to balance the patient's social context. Therefore, it is logical to explore the hypothesis that Ayurvedic treatments will positively affect the patient's perceived social support and understanding of the social world more generally.

Panchakarma is one of the prominent Ayurvedic tools used to restore balance to the body. Through the use of herbalized oils, body treatments (including oil massage), steam therapy, herbal paste therapy, nasal therapy, vomiting therapies, enema therapy, and purgation therapies, Panchakarma is designed to allow the body to rid itself of wastes that have accumulated and lodged in the body, creating blockages in the intelligent flow of the various systems, including the circulatory, nervous, and digestive systems. Once this cleansing process is complete, the body can resume its natural functioning without interference.

To make Panchakarma more accessible to the American audience, specific theoretically driven modifications to the traditional protocol were made at Kripalu, and were directed by the author and certified Ayurvedic practitioner. Specifically, the program was made shorter and some of the techniques were removed. Traditional Panchakarma is usually done in a retreat or even a hospital setting in India over the course of a minimum of 2 weeks, more regularly 4 weeks. Two very common protocols include vomiting (or "*vamana*") and blood letting (or "*rakta moksha*"), depending on the imbalances found. Americans have an aversion to vomiting, are not used to this as a healing protocol, and are usually unwilling to participate in this treatment. Another treatment, *Rakta moksha*, is done with live leeches for which there is a similar aversion in the U.S. All other protocol elements, such as massage, enemas, and dietary changes, were retained and have been used successfully. The timeline of the Kripalu Panchakarma program is as follows:

1. At least 3 weeks before the retreat date, patrons of Kripalu's Panchakarma program engage in a preretreat phone consultation with an Ayurvedic practitioner in which diet and lifestyle behaviors are reviewed. Patrons are guided to modify their diet and begin taking commonly available herbal supplements.
2. The retreat portion of the treatment is conducted at Kripalu Center and includes individual assessments, Ayurvedic massage treatments, a cleansing diet, medicated enemas, group discussions on stress reduction, a personalized yoga session, and a cooking class. This retreat portion lasts 5 days.
3. Participants then return home to complete the last 2 weeks of the cleanse, with lifestyle recommendations to maintain balance for the long term.

We hypothesized that the experience of Panchakarma would improve the participant's sense of perceived social support immediately following the program. Second, the personalized and tailored program should improve the subjects' perceived and reported ability to manage their health. Third, the program should improve their overall sense of well-being. Last, we were curious if any positive effects would be maintained after the program was completed and the individuals returned to their usual life context. In order to gather preliminary data on potential mechanisms of change, we also collected measurements of psychological health (e.g., anxiety with the Beck Anxiety Inventory[22]), physical measurements (e.g., general health with the SF-12 Health Survey[23]), and psychosocial changes (e.g., Bandura's single item of self-efficacy[22]).

### METHODS

The protocol was reviewed and approved by the first author's institutional review board. All subjects received and signed informed consent. In executing this descriptive observational clinical study, we kept in mind the importance of not interrupting the therapeutic process the program was designed to elicit. In this way, naturalistic data were captured and the healing interests of the subjects were protected.

Potential subjects were first told about the study by the director of the Panchakarma program (HG). If the potential subject expressed interest in participating in the study, the director gave the subject the study director's (LC) contact information to being the screening process. All subjects who expressed interest and the ability to finish the protocol were included. No subject was excluded.

When the subject arrived onsite to begin the program, one of the authors (LC) met with her (all of the subjects were women) to review the study in greater depth and to conduct the informed consent process. The baseline measurement packet was administered at this time. Paper and pencil measurements administered to the subjects are three points in time: upon arrival at Kripalu, on the last day of the 5-day program, and 3 months after the completion of the program. This manuscript focuses on the results of these psychosocial and general health instruments (see Table 1).

### Measurements

The *Health-Promoting Lifestyle Profile II*[24] is a 52-item behavior rating scale. It uses a four-point response format to record the frequency of self-reported health-promoting behaviors. It taps into the domains of health responsibility, physical activity, nutrition, spiritual growth, interpersonal relations, and stress management. Questions are asked as frequencies (Never, Sometimes, Often, Routinely) of behaviors. For example, questions include "Take some time for relaxation each day" and "Eat 2–4 servings of fruit each day". It has been used frequently in published work, and has good reliability and validity.

**TABLE 1**
**Selected Measurements Utilized in the Panchakarma Project**

| Scale | Baseline to Post-Treatment Mean Difference | Baseline to 3-Months Post-Treatment Mean Difference |
|---|---|---|
| Lifestyle Profile II[24] | –12.4*** | –14.6* |
| Quality of Life SF-12 Health Survey[23] | 0.833 (ns) | 0.57 (ns) |
| Self-efficacy[22] | 0.42 (ns) | 1.22** |
| Beck Anxiety Inventory[31] | 5.67 (ns) | 0.22* |
| Interpersonal Support[25,26] | –4.3*** | –4.2*** |
| Sarason Social support[27] | 3.15*** | 3.0** |
| Stress[28,29] | 7.56*** | 1.33 (ns) |

Subjects filled out the entire packet at each of the three data collection points.

Significance of differences: *$p \leq 0.1$, **$p \leq 0.05$, ***$p \leq 0.01$, ns = nonsignificant.

The *SF-12*[23] is a self-report measure of quality of life, or general health and function, with good reliability and validity. A single measure of *self-efficacy*[22] was used to measure the subject's belief in her own ability to resolve her symptoms with Ayurvedic techniques. The *Beck Anxiety Inventory*[22] is a 21-item measure, each item describing a common symptom of anxiety. It is well represented in the literature and has good psychometric properties.

The *Interpersonal Support Evaluation List*[25,26] is a 14-item scale that measures how sure the subject is that he/she will receive social support across a variety of circumstances. A modified version of the *Sarason Social Support Questionnaire*[27] was also used to measure respondents' number of social supports and the degree of satisfaction with them. This six-item measure records subjects' perception of the three main types of supportive social interactions: emotional, informational, instrumental. We chose to use two measures of social support; each measures social support in slightly different manners. The former uses specific examples and the latter asks questions in a more general manner. Social support is one of our key constructs and we wanted to ensure that we provided adequate opportunity in the study to capture potential changes in it.

The *Perceived Stress Scale*[28,29] is a 14-item measure that asks about the frequency with which stress is felt in different areas of the individual's life. It is well published with good psychometric properties.

### Statistical Analysis

The SPSS data collection system[30] was used for all data management and analysis. Outcomes were calculated from paired Student's t-tests of difference in means, testing the hypothesis that means were not significantly different per measure over time. Baseline was compared to outcomes after the 5-day retreat. The significance of change over a longer term was considered by comparing means at baseline to means at 3 months postprogram.

### RESULTS

Data collection occurred from February to November of 2006 and included four sessions of the Panchakarma program. Sessions generally host four to eight participants. The initial study sample of 20 women had an age range of 27–54 years. Two were of Asian descent and the rest were Caucasian. Most had graduated from a 4-year college or university, and reported an annual income of $50,000 or more. The full sample completed the post-treatment measurement and 12 women completed the 3-month follow-up.

Table 1 lists the scales used along with mean group differences over time and significance level of any changes over time. Measurement can be broken into behavioral and psychosocial measures. The two behavioral measures, the Lifestyle Profile (which measures reported adherence to positive lifestyle practices) and self-efficacy (which measures belief in one's ability to make positive health changes with these practices), both showed significant improvement at 3 months postintervention. Anxiety decreased significantly at 3 months post-treatment, but not immediately after treatment. As we were particularly interested in social support and how it may operate through the mechanism of stress, we included multiple measures of these. All three improved post-treatment, but only the social measures held significant change to 3 months postintervention.

### DISCUSSION

One challenging aspect of measuring such a cleansing program is that, theoretically, subjects may feel worse on the way to feeling better. Ayurveda addresses this as the body's cleansing mechanism and other CAM systems label such reactions as "healing crises". Given this, the researchers were surprised that there were no serious side effects reported and a low level of reported mild side effects, such as gastrointestinal upset or brief insomnia. At baseline, 21% of subjects reported mild side effects, which most likely represent the effects of the preprogram dietary changes and the experience of everyday life. The moderate level of reported side effects rose insignificantly to 26% after 5 days at the retreat. While this result is positive, we were disappointed that our main hypothesis was not supported; there were also no significant changes, positive or negative, in general symptoms as measured by the SF-12[23]. There are many possible explanations for this: a true lack of benefit to overall quality of life, the negative effects of the cleansing process were balanced by any positive effects, or the self-report measurement was not sensitive in this sample. We were also surprised that anxiety decreased significantly at 3 months post-treatment, but not immediately after treatment; this could be an indication of the situational anxiety caused by the deep cleansing program, and lack of ability to rely on home-based behavior patterns and comfortable routines.

While we did not find evidence that overall quality of life was improved through the use of Panchakarma, as a program of behavior change, our preliminary results suggest that the complex intervention may be effective in assisting one's actual and expected adherence to new and healthier behavior patterns. That is, with statistical significance, following the intervention, the Lifestyle Profile II showed a greater frequency of positive patterns and subjects reported greater self-efficacy in using Ayurveda to promote positive health changes.

It also appears that that perceived social support might be part of the mechanism of these changes. With a larger sample and adequate controls, we could model social support as a mediator or modifier of behavior change. Yet it may be more appropriate to consider the social world as a necessary part of a holistic intervention. While social factors may mediate one's ability to achieve a certain outcome, considering such changes exclusively in a linear manner, at least in the beginning of an investigation, would impede our ability to see how factors are related to each other. Research in this area has indicated that the addition of social support, even as a piece of a larger intervention, may not be effective[19]. Perhaps an important mechanism in complex CAM systems is the very thing that subjects report enjoying most — the patient is considered as a whole, in context[7]. Social support is not an added factor, but an emergent quality of the system. This is supported by other research that shows that behavior change programs are more effective the more relevant the messages are or how well the program is incorporated into the person's regular life[32]. Behavior change programs work better when the program is made salient to the subject. Improving perceived social support might be a way to improve salience and social support may be easily influenced through holistic systems.

If this is true, then it points to new questions in the area of health behavior research. One frustration to health behavior scientists is that, generally, important aspects of subjects' social world are nonmodifiable. Many of our health risks are affected by our social determinants — race, gender, education — factors that are not easily modifiable. Yet the results of these social determinants — anxiety, low self-efficacy — may be modifiable in the context of complex CAM medical interventions for the very reason that such interventions work inherently on the level of the subject's social world.

But how do we know that we modified perceived social support and did not just add new social connections? While not a main study outcome, we wanted to consider this question and thus we also measured the subjects' reported social network index[33] at the same three data collection points. The social network index is a structural measure of the subject's social contacts and the use of those contacts. No significant reported changes were found following Panchakarma in this structural measure. This is interesting because it supports the idea that perceived social support could be increased without actual structural changes in the social network. The way that the subject thinks about his/her social world changes, even if no new individuals are added; it is a perceptual and phenomenological change, not a structural change. Adding supportive people may help, but this study suggests that changing the individual's perceptions, without adding people, may also be important.

Much research has shown that perceptual factors, such as quality of life and expectation about health, are predictive of overall health[32,34]. Holistic health interventions may offer insights on how to modify lived experience. In such a holistic world view, lived or phenomenological experiences are factors or symptoms that can be treated. This project also included the collection of three qualitative interviews conducted prior to the retreat, immediately after, and 3 months postretreat. The interviews investigated the subject's health history, health practices, expectations of treatment, and experiences with the Panchakarma program. A future step for this project is to analyze the qualitative interview data in order to add a phenomenological understanding of this process, and perhaps to link phenomenology with standardized outcomes. If the qualities of lived experience are related to later disease outcomes, then examining and modifying such qualities may be helpful in disease prevention. This idea is utilized in other well-studied health sciences[35,36], so learning more about how Ayurveda models such relationships will likely add to our scientific understanding of the mechanisms of health and healing.

### Limitations

Although these results are encouraging, a significant limitation is the generalizability of the sample. This is a self-selected population and data were not collected on the individuals who dropped out, leaving us unsure if those that remained in the study had more positive experiences. Future work would be strengthened by the addition of a control group, the addition of selected biomarkers of health status, and further exploration of who dropouts are. Still, our initial preliminary look at this program is promising, both in terms of feasibility, but also in that we achieved statistical significance for the measures selected. Also, our use of a long-term follow-up allowed us to see that the positive effects achieved during the program were maintained 3 months after the program was completed and the individuals returned to their usual life context.

### ACKNOWLEDGMENT

The research was supported by Grant Number 1K24 AT004095 from the National Center for Complementary and Alternative Medicine (NCCAM).

## References

1. NCCAM Backgrounder. "Ayurvedic Medicine: an Introduction". July, 2008. Accessed November 28, 2008. http://nccam.nih.gov/health/ayurveda/.
2. Sharma, H., Candela, H.M., Singh, G., and Bassist, G.(2007) Utilization of Ayurveda in health care: an approach for prevention, health promotion, and treatment of disease. Part 2--Ayurveda in primary health care. *J. Altern. Complement. Med*. **13(10),** 1135–1150.
3. Sharma, H., Chandola, H.M., Singh, G., and Basisht, G. (2007) Utilization of Ayurveda in health care: an approach for prevention, health promotion, and treatment of disease. Part 1--Ayurveda, the science of life. *J. Altern. Complement. Med*. **13(9),** 1011–9.
4. Jonas, W.B. (2002) Evidence, ethics, and the evaluation of global medicine. In *The Role of Complementary and Alternative Medicine: Accommodating Pluralism*. Callahan, D., Ed. Georgetown University Press. pp. 122–143.
5. Walach, H. et al. (2006) Circular instead of hierarchical: methodological principles for the evaluation of complex interventions. *BMC Med. Res. Methodol*. **6,** 29.
6. Campbell, M. et al. (2000) Framework for design and evaluation of complex interventions to improve health. *BMJ* **321,** 694–696.
7. Paterson, C. and Britten, N. (2004) Acupuncture as a complex intervention: a holistic model. *J. Altern. Complement. M*[illegible] 791–801.
8. Paterson, C. (2006) Measuring changes in self-concept: a qualitative evaluation of outcome questionnaires in people [illegible] acupuncture for their chronic health problems. *BMC Complement. Altern. Med*. **6,** 7.
9. Kiecolt-Glaser, J.K., Fisher, L.D., Ogrocki, P., Stout, J.C., and Speicher, C.E. (1987) Marital quality, marital disruption and immune function. *Psychosom. Med*. **49,** 13–34.
10. Thomas, P.D., Goodwin, J.M., and Goodwin, J.S. (1985) Effect of social support on stress-related changes in cholesterol levels, uric acid level and immune function in an elderly sample. *Am. J. Psychiatry* **142,** 735–737.
11. Pressman, S., Cohen, S., Miller, G.E., Barkin, A., Rabin, B.S., and Treanor, J. (2005) Loneliness, social network size and immune response to influenza vaccination in college freshmen. Health Psychol. **24(3),** 297–306.
12. Cohen, S. (2004) Social relationships and health. *Am. Psychol*. **59,** 676–684.
13. Uchino, B.N., Cacciopo, J.T., and Kiecolt-Glaser, J.K. (1996) The relationship between social support and physiological processes: a review with emphasis on underlying mechanisms and implications for health. *Psychol. Bull*. **119,** 488–531.
14. Wright, R.J., Cohen, S., Wand, M., and Gold, D.R. (2004) Chronic caregiver stress and IgE expression, allergen-induced proliferation, and cytokine profiles in a birth cohort predisposed to atopy. *J. Allergy Clin. Immunol*. **113,** 1051–1057.
15. Miller, G.E., Cohen, S., and Ritchey, K.A. (2002) Chronic psychological stress and the regulation of pro-inflammatory cytokines: a glucocorticoid-resistance model. *Health Psychol*. **21(6),** 531–541.
16. Cohen, S., Doyle, W.J., et al. (2004) Childhood socioeconomic status and host resistance to infectious illness in adulthood. *Psychosom. Med*. **66,** 553–558.
17. Berkman, L. and Kawachi, I. (2000) *Social Epidemiology*. Oxford University Press, New York.
18. Glymour, M.M., Weuve, J., Fay, M.E., Glass, T., and Berkman, L.F.(2008) Social ties and cognitive recovery after stroke: does social integration promote cognitive resilience? *Neuroepidemiology* **31(1),** 10–20.
19. Glass, T.A., Berkman, L.F., Hiltunen, E.F., Furie, K., Glymour, M.M., Fay, M.E., and Ware, J. (2004) The Families In Recovery From Stroke Trial (FIRST): primary study results. *Psychosom. Med*. **66(6),** 889–897.
20. Taylor-Piliae, R.E., Haskell, W.L., Waters, C.M., and Froelicher, E.S. (2006) Change in perceived psychosocial status following a 12-week Tai Chi exercise programme. *J. Adv. Nurs*. **54(3),** 313–329.
21. Kennedy, J.E., Abbott, R.A., and Rosenberg, B.S.(2002) Changes in spirituality and well-being in a retreat program for cardiac patients. *Altern. Ther. Health Med*. **8(4),** 64–66, 68–70, 72–73.
22. Bandura, A. (1977) Self-efficacy: toward a unifying theory of behavioral change. *Psychol. Rev*. **84(2),** 191–215.
23. Ware, J.E., Kosinski, M., and Keller, S.D. (1996) A 12-Item Short-Form Health Survey: construction of scales and preliminary tests of reliability and validity. *Med. Care* **34(3),** 220–233.
24. Walker, S.N., Sechrist, K.R., and Pender, N.J. (1987) The health-promoting lifestyle profile: development and psychometric characteristics. *Nurs. Res*. **36(2),** 76–81.
25. Cohen, S. and Hoberman, H. (1983) Positive events and social supports as buffers of life change stress. *J. Appl. Soc. Psychol*. **13,** 99–125.
26. Cohen, S., Mermelstein, R., Kamarck, T., and Hoberman, H. (1985) Measuring the functional components of social support. In *Social Support: Theory, Research, and Application*. Sarason, I.G. and Sarason, B.R., Eds. Martinus Nijhoff, The Hague.
27. Sarason, I.G., Sarason, B.R., Shearin, E.N., et al. (1987) A brief measure of social support: practical and theoretical implications. *J. Soc. Personal Relations* **4,** 497–510.
28. Cohen, S. and Williamson, G. (1988) Perceived stress in a probability sample of the U.S. In *The Social Psychology of Health: Claremont Symposium on Applied Social Psychology.* Spacapam, S. and Oskamp, S., Eds. Sage, Thousand Oaks, CA.
29. Cohen, S., Kamarck, T., and Mermelstein, R. (1983) A global measure of perceived stress. *J. Health Soc. Behav*. **24,**

385–396.
30. SPSS for Windows, Rel. 15.0. 2006. SPSS, Inc. Chicago.
31. Beck, A.T. and Steer, R.A. (1993) *Beck Anxiety Inventory Manual*. Psychological Corporation, San Antonio, TX.
32. Glanz, K., Rimer, B.K., and Lewis, F.M. (2002) *Health Behavior and Health Education: Theory, Research, and Practice*. Jossey-Bass, San Francisco.
33. Cohen, S., Doyle, W.J., Skoner, D.P., Rabin, B.S., and Gwaltney, J.M., Jr. (1997) Social ties and susceptibility to the common cold. *JAMA* **277,** 1940–1944.
34. Rosenstock, I.M.(1988) Adoption and maintenance of lifestyle modifications. *Am. J. Prev. Med.* **4(6),** 349–352.
35. Ventegodt, S., Clausen, B., Nielsen, M.L., and Merrick, J. (2006) Clinical holistic health: advanced tools for holistic medicine. *TheScientificWorldJOURNAL* **6,** 2048–2065**.**
36. Galland, L. (2006) Patient-centered care: antecedents, triggers, and mediators. *Altern. Ther. Health Med.* **12(4),** 62–70.
